# Traumateamaktivierung nach Verkehrsunfällen – Auswirkung der Indikationsänderungen in der S3-Leitlinie Polytrauma/Schwerverletzten-Behandlung

**DOI:** 10.1007/s00113-025-01636-0

**Published:** 2025-09-29

**Authors:** Bastian Brune, Max Bittner, Fabian Haut, Maximilian Wolf, Sascha Keil, André Nohl, Frank Herbstreit, Christian Waydhas, Lars Becker, Marcel Dudda

**Affiliations:** 1 Klinik für Unfall‑, Hand- und Wiederherstellungschirurgie, Universitätsklinikum Essen, Hufelandstr. 55, 45147 Essen, Deutschland; 2 Ärztliche Leitung Rettungsdienst, Feuerwehr Essen, Essen, Deutschland; 3 Feuerwehr Essen, Essen, Deutschland; 4 Ärztliche Leitung Rettungsdienst, Feuerwehr Oberhausen, Oberhausen, Deutschland; 5 Zentrum für Notfallmedizin, BG-Klinikum Duisburg, Duisburg, Deutschland; 6 Klinik für Anästhesiologie und Intensivmedizin, Universitätsklinikum Essen, Essen, Deutschland; 7 Klinik für Unfallchirurgie und Orthopädie, BG-Klinikum Duisburg, Duisburg, Deutschland

**Keywords:** Verkehrsunfall, Triage, Fehleinschätzung, Versorgungsqualität, Krankenhausverweildauer, Traffic accident, Triage, Diagnostic errors, Quality of care, Length of stay

## Abstract

**Hintergrund:**

Mit der Aktualisierung der S3-Leitlinie „Polytrauma/Schwerverletzten-Behandlung“ im Jahr 2023 wurden die Kriterien zur Traumateamaktivierung (TTA) überarbeitet. Dabei wurden unfallmechanische Kriterien weitgehend entfernt. Die Auswirkungen der Leitlinienänderungen auf die Versorgung in der zentralen Notaufnahme (ZNA), auf die Über- und Untertriage nach Verkehrsunfällen sowie auf die Krankenhausverweildauer wurden bisher nicht nachuntersucht.

**Fragestellung:**

Ziel der Arbeit war, die Auswirkung der Leitlinienaktualisierung auf die Behandlung in einer Zentralen Notaufnahme eines überregionalen Traumazentrums (ÜTZ) zu bewerten.

**Methoden:**

In einer monozentrischen, prospektiven Beobachtungsstudie wurden über 2 Jahre (Prä- vs. Post-Leitlinienaktualisierung) alle Patient:innen nach motorisierten Verkehrsunfällen in einem ÜTZ erfasst. Primäre Endpunkte waren Häufigkeit und Adäquatheit der TTA sowie die Verteilung der Behandlungsorte. Sekundärer Endpunkt war die Verweildauer.

**Ergebnisse:**

Insgesamt wurden 1438 Fälle ausgewertet. Die Anzahl der rot triagierten Patient:innen sank signifikant (257 vs. 157; *p* < 0,001). Die Rate an Übertriage bei TTA sank (27,6 % vs. 21 %, *p* < 0,01), ohne signifikante Zunahme der Untertriage. Mortalität und Verweildauer unterschieden sich nicht signifikant zwischen beiden Gruppen.

**Schlussfolgerung:**

Die Änderung der TTA-Kriterien führte zu einer deutlichen Abnahme der TTA bei gleichbleibender Versorgungsqualität. Eine signifikante Zunahme von Fehleinschätzungen (Untertriage) konnte nicht festgestellt werden. Die Änderung der TTA-Kriterien kann zu einer ressourcenschonenden Behandlung ohne -gefährdung beitragen.

**Graphic abstract:**

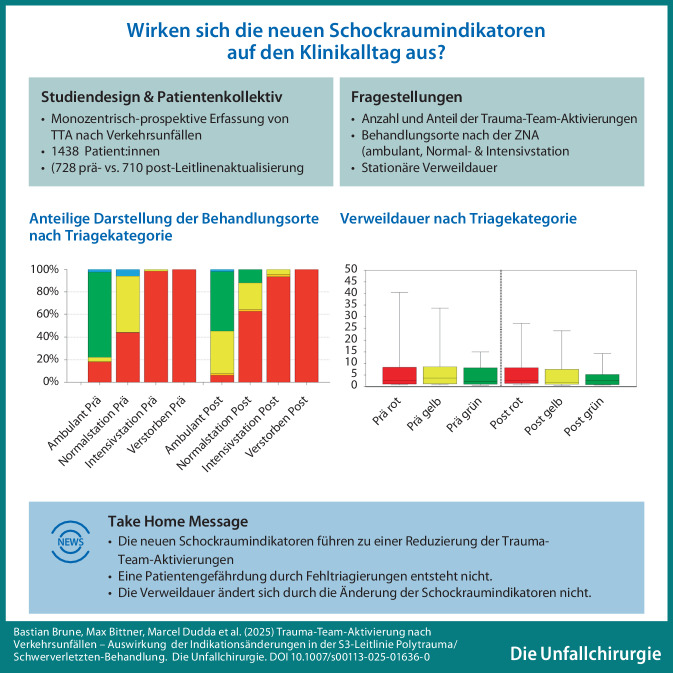

## Abstract (en)

**Background:**

With the 2023 revision of the German S3 guideline “Polytrauma/severely injured patient care” the criteria for trauma team activation (TTA) were revised. Accident mechanism-based criteria were largely eliminated. The impact of these changes on emergency department (ED) management, over- and undertriage following traffic accidents, and hospital length of stay has not yet been systematically evaluated.

**Objective:**

The aim of this study was to assess the effects of the guideline update on patient treatment in the emergency department of a level I trauma center.

**Methods:**

In a prospective, single-center observational study, all patients presenting after motor vehicle accidents were recorded over a 2-year period (pre- vs. postguideline update). Primary endpoints were the frequency and appropriateness of TTA as well as the distribution of treatment locations. Secondary endpoint was hospital length of stay.

**Results:**

A total of 1438 cases were analyzed. The number of red-triaged patients decreased significantly (257 vs. 157; *p* < 0.001). The overtriage rate among TTA patients declined (27.6% vs. 21%, *p* < 0.01) without a significant increase in undertriage. Mortality and hospital length of stay did not differ significantly between groups.

**Conclusion:**

The revision of TTA criteria led to a marked reduction in trauma team activations while maintaining quality of care. No significant increase in undertriage was observed. The updated TTA criteria may support resource-efficient patient care without compromising safety.

## Einleitung

Im Rahmen der Aktualisierung der S3-Leitlinie Polytrauma/Schwerverletztenbehandlung wurden die Alarmierungskriterien zur Behandlung im Schockraum angepasst [1, 2]. Neben der Anpassung der ehemaligen A‑ und B‑Kriterien zur Traumateamaktivierung (TTA) an die internationale Nomenklatur zu sog. HRSI- („high risk for severe injury“) und MRSI-Kriterien („moderate risk for severe injury“) wurden erstmalig altersspezifische Besonderheiten geriatrischer Patient:innen betont und in den Indikationskatalog zur TTA aufgenommen. Angaben zum Unfallmechanismus, wie z. B. Verformungen der Fahrzeugkarosserie oder Fahrtgeschwindigkeitsveränderungen, wurden aufgrund der hohen Übertriagierungsanteile nicht mehr berücksichtigt [1, 3]. Lediglich die Ejektion aus einem Fahrzeug und eine Verletzung mit Fraktur langer Röhrenknochen sind als unfallmechanismusbezogene Gründe zur TTA verblieben.

Vor der vorangegangenen Leitlinienaktualisierung (LA) 2015 erfolgte die Zuweisung von ca. einem Drittel der Schockraumpatient:innen in ein überregionales Traumazentrum (ÜTZ) aufgrund des Unfallmechanismus [4]. Auch wenn eine Einbindung des Unfallmechanismus in die Triage den positiv prädiktiven Wert einer Schockraumalarmierung steigern kann, führt eine alleinig auf den Mechanismus bezogene Alarmierung zu einer Übertriage von 60–70 % [5]. In einer multizentrischen Befragung in einem einmonatigen Beobachtungszeitraum konnte diese nach der LA auf 24 % gesenkt werden [6].

In der aktuellen Studie sollen prospektiv die Auswirkungen der Änderung über ein Jahr vor und nach dieser in einem ÜTZ nach Verkehrsunfällen bewertet werden. Ziel der Untersuchung war es, prospektiv den Einfluss der Aktualisierung der S3-Leitlinie „Polytrauma/Schwerverletzten-Behandlung“ der Deutschen Gesellschaft für Unfallchirurgie e. V. (DGU) aus dem Jahr 2022 auf die Versorgungsstruktur und die Zuweisung von Patient:innen nach Verkehrsunfällen mit Kraftfahrzeugen zu analysieren. Insbesondere wurden hier die Auswirkungen der veränderten Kriterien zur rettungsdienstlichen TTA mit folgender Triage in der ZNA und Behandlung in der aufnehmenden Zielklinik betrachtet.

## Material und Methoden

### Studiendesign

Es handelt sich um eine monozentrische, prospektive Beobachtungsstudie mit einem Vergleich vor und nach der Aktualisierung der S3-Leitlinie Polytrauma/Schwerverletzten-Versorgung. Eine Intervention wurde im Rahmen der Analyse nicht vorgenommen. Die Datenerhebung und -bewertung wurde an einem ÜTZ und Referenzzentrum für Kindertraumatologie, mit Zulassung zum Schwerstverletztenverfahren (SAV) der Deutschen Gesetzlichen Unfallversicherung (DGUV) durchgeführt. Das Zentrum liegt in einem urbanen Ballungsraum und nimmt als Primär- und Sekundärversorger Patient:innen mit überregionalem Einzugsgebiet mehrerer Rettungsdienste auf.

### Primäre und sekundäre Zielparameter

Zur Auswertung der Beobachtung wurden folgende primäre Zielparameter definiert:

- Anzahl und Verhältnis der TTA und übrigen Behandlungen in der ZNA,
- Behandlungsort (ambulant, Normalstation, Intensivstation) nach Initialer Triage.

Sekundäre Zielparameter waren:

- Verweildauer von Patient:innen in Abhängigkeit von der initialen Triage.

### Studienkollektive

Es wurden über einen Zeitraum von 2 Jahren fortlaufend alle Patient:innen nach Verkehrsunfällen mit Beteiligung eines Kraftfahrzeugs erfasst und in Bezug auf den Tag der Veröffentlichung der neuen S3-Leitlinie „Polytrauma/Schwerverletzten-Behandlung“ am 14.02.2023 eingeteilt. Es wurden die Gruppen Prä- (Prä-LA) (14.02.2022–13.02.2023) und Post-Leitlinienaktualisierung (Post-LA; 14.02.2023–13.02.2024) gebildet. Um die Behandlung zu bewerten, wurden die Patient:innen außerdem nach Triagierungskategorien aufgeteilt. Einer rettungsdienstlichen Indikationsstellung zur TTA erfolgte im klinischen Setting stets die Dringlichkeitseinstufung „Sofort“ (rot) im Manchester-Triagesystem (MTS; Tab. 1). Falls rettungsdienstlich keine TTA initiiert wurde, erfolgte eine Kategorisierung durch das Pflegepersonal in der ZNA. Um die Triage zu bewerten, wurde die Untertriage als Behandlung in der ZNA ohne TTA mit anschließendem Aufenthalt auf einer Intensivstation gewertet. Als Übertriage wurde eine Behandlung in der ZNA mit TTA, jedoch ohne erforderliche intensivmedizinische Behandlung gewertet.

**Tab. 1:** Kategorien im Manchester-Triagesystem nach Mayerhofer et al. [15].

Dringlichkeit	Sichtungskategorie	Maximale Wartezeit (min)
Sofort	Rot	0
Sehr dringend	Orange	10
Dringend	Gelb	30
Normal	Grün	90
Nicht dringend	Blau	120

### Einschlusskriterien

Eingeschlossen wurden alle Patient:innen, die nach einem Verkehrsunfall mit einem Kraftfahrzeug (PKW, LKW, Bus, Kraftrad) in der zentralen Notaufnahme (ZNA) rettungsdienstlich vorgestellt wurden oder sich eigenständig vorstellten. Zu diesen zählten neben Insassen von Kraftfahrzeugen auch Fußgänger:innen, Radfahrer:innen oder anderweitig am Verkehr beteiligte Personen, die von solchen Fahrzeugen angefahren worden waren. Ebenso wurden Primärzuweisungen und Sekundärverlegung aus anderen Krankenhäusern eingeschlossen.

Die Behandlungsdaten wurden aus der klinischen Dokumentation (Arztbriefe, Rettungsdienstprotokolle, der Triagehistorie und dem Krankenhausinformationssystem [KISS]) entnommen und flossen im Falle eines Einschlusses in das TraumaRegister der DGU® (TR-DGU®) in selbiges ein.

Der Bedarf einer intensivmedizinischen Behandlung wurde über die erste stationäre Behandlung, also nach der ZNA bzw. dem Operationssaal (OP), definiert. Komplikative Verläufe, die erst in einem späteren Behandlungsintervall eine intensivmedizinische Behandlung erforderlich machten, wurden nicht betrachtet.

### Ausschlusskriterien

Von der Beobachtung ausgeschlossen wurden Patient:innen, bei denen zwischen Unfallzeitpunkt und Vorstellung mehr als 48 h vergangen waren. Auch wurden Patient:innen ausgeschlossen, bei denen der genaue Zeitraum des Verkehrsunfalls oder der Unfallmechanismus unklar blieben.

## Datenerhebung

In der prospektiven Datenerhebung wurden alle ZNA-Berichte aus dem Erfassungszeitraum gesichtet. Im Falle eines motorisierten Verkehrsunfalls wurden die Fallnummern der eingeschlossenen Patient:innen zur Datensatzverknüpfung mit der fallbezogenen Dokumentation genutzt (Tab. 1). Die Behandlung wurde incl. der Triage im MTS in ERPath (epias GmbH, Idstein, Deutschland) dokumentiert.

Für Patient:innen, die in das TR-DGU® aufgenommen wurden erfolgte die Berechnung des Injury Severity Scores (ISS). Für Patient:innen, die nicht ins TR-DGU® aufgenommen wurde, wurde ein ISS < 16 angenommen. Im Falle eines relevanten ISS ≥ 16 wäre durch die klinikinterne Qualitätssicherung eine Eingabe ins TR-DGU® erfolgt und somit ein ISS verfügbar.

### Ethik

Der bei der Ethikkommission der Universität Duisburg-Essen eingereichte Antrag wurde positiv beschieden (23-11259-BO).

## Statistische Analyse

Die Statistische Auswertung erfolgte mittels Mann-Whitney-U-Tests und χ^2^-Test in SPSS® (IBM®, Armonk, NY, USA). Berechnet wurden jeweils Mittelwert und Standardabweichung. Als statistisch signifikant wurde ein *p* ≤ 0,05 definiert.

## Ergebnisse

Insgesamt wurden 1438 Patient:innen in die Auswertung eingeschlossen. Davon wurden 728 Fälle der Gruppe Prä-LA und 710 Fälle der Gruppe Post-LA zugeordnet. 55 (36 Prä-LA, 19 Post-LA) Patient:innen wurden aufgrund unzureichender Dokumentation des Unfallzeitpunktes oder verspäteter Vorstellung ausgeschlossen. Demographisch zeigten sich keine signifikanten Unterschiede in den beiden Gruppen (Tab. 2).

**Tab. 2:** Demographie

	Prä-LA (%)	Post-LA (%)
≤ 17 Jahre	94 (12,9)	106 (14,9)
W	36 (4,9)	33 (4,5)
M	58 (8)	73 (10,2)
18–69 Jahre	594 (81,6)	563 (79,3)
W	234 (32,1)	251 (35,2)
M	360 (49,5)	318 (44,7)
≥ 70 Jahre	40 (5,5)	41 (5,8)
W	17 (2,3)	13 (1,7)
M	23 (3,2)	28 (3,7)

*W* weiblich, *M* männlich, *LA* Leitlinienaktualisierung

In der Prä-Leitlinienkohorte wurden 257 Patient:innen rot triagiert und resultierend eine TTA vorgenommen. 4 Patient:innen wurden orange und 467 Patient:innen gelb oder weniger dringlich, also blau oder grün, kategorisiert (gelb: 143, grün: 314; blau: 10; Abb. 1). In der Post-Leitlinienkohorte wurden 157 Patient:innen rot, 12 Patient:innen orange und 541 Patient:innen gelb oder weniger dringlich triagiert (gelb: 234, grün: 297, blau: 10).

**Abb. 1:**
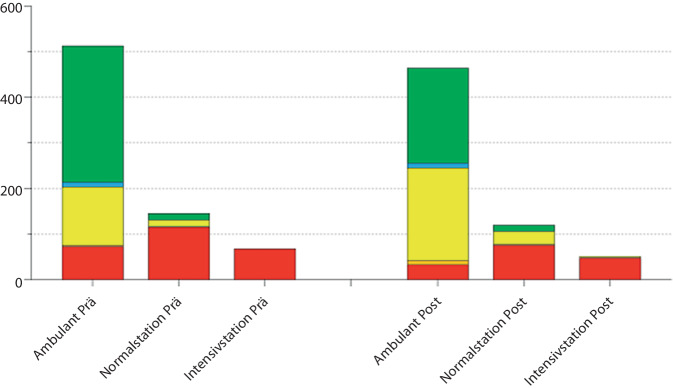
Absolute Fallzahlen nach Triagierungskategorien in den Zeiträumen vor und nach Leitlinienaktualisierung (LA)

Es zeigt sich bereits im Gesamtdatenvergleich, ohne einen direkten Einfluss der LA auf die MTS, ein signifikanter Rückgang der Anzahl rot triagierter Personen (257 vs. 157, χ^2^, *p* < 0,001).

### Patient:innen mit primärer TTA

**Abb. 2:**
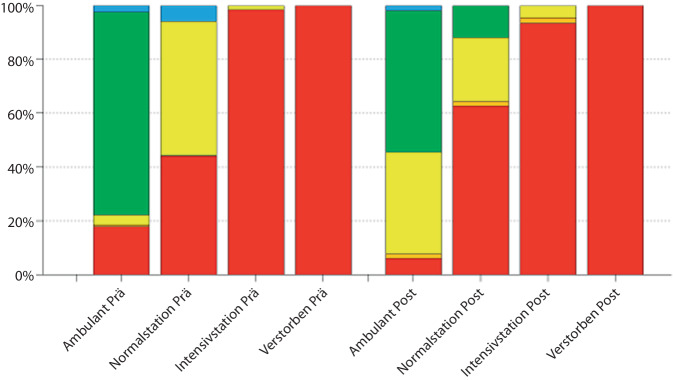
Relative Fallzahlen nach Triagierungskategorien in den Zeiträumen vor und nach Leitlinienaktualisierung (LA)

Vor der LA wurden 65 von 257 (25,3 %) der rot triagierten Patient:innen nach der Versorgung in der ZNA oder der ersten operativen Therapie auf einer Intensivstation behandelt (Abb. 2). Nach Aktualisierung der Leitlinie waren es 43 von 157 Patient:innen (27,4 %). Dies stellt keinen signifikanten Unterschied dar.

Ebenfalls nicht signifikant unterscheiden sich die Anzahl und Anteil der Patient:innen, die vor und nach Aktualisierung der Leitlinie auf einer Normalstation behandelt wurden. Vor Änderung der Leitlinie waren es 114 von 257 (44,4 %), nach Aktualisierung der Leitlinie waren es 74 von 157 Patient:innen (47,1 %).

Signifikant (χ^2^, *p* = 0,002) gingen hingegen Anzahl und Anteil der rot triagierten Patient:innen zurück, die nach einer primären TTA ambulant verblieben sind. Dies waren vor der Änderung der Leitlinie 71 von 257 (27,6 %). Nach LA waren es lediglich 33 von 157 (21 %).

### Patient:innen ohne primäre TTA

Insgesamt haben die Anzahl und der Anteil der Patient:innen, die nicht primär im Schockraum vorgestellt wurden, signifikant (χ^2^, *p* < 0,001) zugenommen. Waren es vor der LA noch 471 von 728 Patient:innen (64,7 %), waren es nach der Aktualisierung der Leitlinie bereits 553 von 710 Patient:innen (77,9 %).

Es konnte keine Zunahme der Patient:innen beobachtet werden, die nicht primär im Schockraum vorgestellt, aber nach notfallmedizinischer Behandlung oder der ersten operativen Therapie in intensivmedizinischer Behandlung verblieben sind. In beiden Kohorten handelte es sich um Einzelfälle. Vor der Aktualisierung der Leitlinie traf dies genau auf einen Fall von 440 (0,2 %) zu. Nach der Änderung der Leitlinie wurden drei Patient:innen von 553 (0,5 %) initial nicht schockraumpflichtigen intensivmedizinisch therapiert.

Der Anteil der nicht über den Schockraum vorgestellten Patient:innen, die auf einer peripheren Station oder Normalstation behandelt wurden, hat nicht signifikant zugenommen. Vor der LA waren es 30 der 471 (6,4 %) Patient:innen, anschließend 44 der 553 (8 %).

### Intensivmedizinische Behandlung und ISS

Einen durchschnittlichen ISS von 28,2 ± 12,4 Punkten (MW + SD) wiesen 46 Patient:innen aus dem Gesamtkollektiv der primär im Schockraum behandelten Patient:innen mit anschließender Behandlung auf einer Intensivstation auf. Der ISS in der Prä-LA (*n* = 27) unterschied sich mit 30 ± 13,7 nicht signifikant von dem der Post-Leitlinienkohorte (*n* = 19, ISS 24 ± 9,2) (*p* = 0,04).

**Abb. 3:**
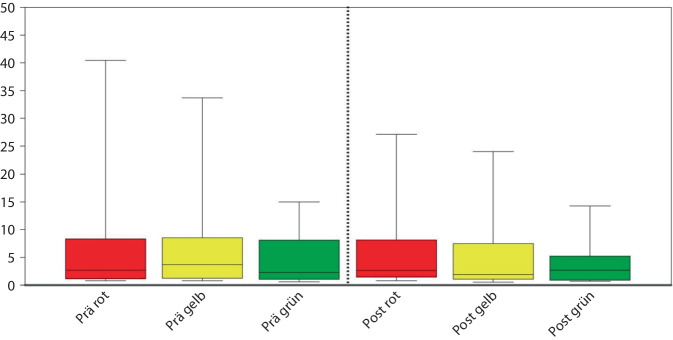
Krankenhausverweildauer nach Triagierungskategorie vor und nach Leitlinienaktualisierung (LA)

Die Verweildauer von Patient:innen mit einem ISS ≥ 16, die über den Schockraum vorgestellt wurden, war in der Gruppe Prä-LA im Durchschnitt 24,81 ± 30,49 Tage. In der Gruppe Post-LA betrug sie im Durchschnitt 15,56 ± 17,42 Tage (*p* = 0,12) (Abb. 3).

Darüber hinaus wurden 3 Patient:innen identifiziert, die nicht primär im Schockraum behandelt wurden. Diese wurden zuvor jeweils gelb triagiert. Die Patient:innen wiesen nach primärer Diagnostik entweder einen ISS von deutlich > 16 auf oder wurden in einem Fall aufgrund von Überwachungsstandards bei pädiatrischen Patient:innen bei einem ISS < 16 intensivmedizinisch betreut.

Alle Patient:innen mit einem ISS ≥ 16 wurden im Anschluss an die Primärversorgung im Krankenhaus auf einer Intensivstation betreut oder verstarben im Verlauf der Schockraumtherapie.

### Mortalität

In beiden Kohorten verstarben jeweils 7 Patient:innen (0,9 % vs. 1,0 %, *p* > 0,05). Diese wurden jeweils rot triagiert und nach TTA im Schockraum behandelt. In Bezug auf die primär im Schockraum therapierten Patient:innen lag die Mortalität bei 2,5 % vs. 4,3 % (*p* > 0,05).

Vor Änderung der Leitlinie verblieben die verstorbenen Patient:innen im Durschnitt 4,9 ± 6,3 Tage in intensivmedizinischer Behandlung. Nach Änderung der Leitlinie verblieben verstorbene Patient:innen im Mittel 2,6 ± 1,2 Tage in intensivmedizinischer Behandlung. Die Behandlungszeiträume der Patient:innen vor Ihrem Versterben unterscheidet sich nicht signifikant. Vor der LA lag der mittlere ISS von Patient:innen, die im Laufe des Krankenhausaufenthalts verstarben, bei 47,6 ± 12,2, während der mittlere ISS nach der Leitlinienäderung bei 30,7 ± 14,1 lag. Diese ISS-Abweichungen sind nicht signifikant.

## Diskussion

Nach Einführung der aktualisierten S3-Polytraumaleitlinie zeigte sich ein signifikanter Rückgang an primär über den Schockraum zugewiesenen Patient:innen, ohne Hinweise darauf, dass es bei tatsächlich schwerverletzten Patient:innen weniger TTA gab. Innerhalb der Gruppe nicht im Schockraum vorgestellter Patient:innen konnte keine verlängerte stationäre Verweildauer oder ein Anstieg intensivmedizinisch versorgter Fälle beobachtet werden. Die Versorgung von Patient:innen außerhalb des Schockraums ohne TTA wirkte sich somit nicht negativ aus.

Eine vergleichbare, aktuelle Arbeit von Wolf et al. [6] beschreibt, basierend auf einen kurzen Beobachtungszeitraum, eine Zuweisungsbegründung von 55,9 % HRSI, 17 und 18,9 % kombinierten Kriterien. So wird trotz Kenntnis der LA immer noch ein relevanter Anteil an Patient:innen auf Grundlage der den Unfallmechanismus betreffenden Kriterien der letzten Leitlinie über den Schockraum vorgestellt. Außerdem erfolgt generell ein relevanter Anteil der Zuweisung weiterhin ohne Bezug auf formelle Kriterien. 8,0 % der Fälle dieser Arbeitsgruppe wiesen weder HRSI-, noch MRSI-Kriterien auf. Die Schockraumzuweisung basierte allein auf der (prä-)klinischen Entscheidung („provider decision“; [6]). Der Vergleich zum eigenen Kollektiv ist aufgrund der isolierten Betrachtung von Verkehrsunfällen und der klinikspezifischen Zusammensetzung der im Schockraum behandelten Patient:innen erschwert.

Durch die Implementierung der neuen Alarmierungskriterien konnte der Anteil der Übertriage innerhalb eines Jahres signifikant reduziert werden, bleibt jedoch mit 72,6 % sowohl weiterhin hoch, als auch höher als in der Arbeit von Wolf et al. [6]. Die bestehende Übertriage kann dadurch erklärt werden, dass die Umsetzung von Leitlinien prozessbedingt zeitlich verzögert stattfindet [7, 8]. Eine weitere Verbesserung der rettungsdienstlich gestellten Indikationen zur TTA erscheint somit im weiteren Zeitverlauf möglich.

### Unter- und Übertriage

Bei der Bewertung der Schockraumalarmierungskriterien ist eine niedrige Untertriage und eine reduzierte Übertriage anzustreben [5, 6, 9–11]. Sowohl eine Übertriage mit einer hohen Bindung von personellen und materiellen Ressourcen bei leicht verletzten Patient:innen, als auch eine personal- und materialsparende Untertriage bei schwerverletzten Patient:innen sollten vermieden werden [1, 12]. Die Übertriage stellt besonders außerhalb der Regelarbeitszeit, aufgrund der eingeschränkt verfügbaren Personalressourcen, eine relevante Belastung dar.

Dennoch formulieren Kühne et al. [13], dass die Übertriage „im Vergleich zur Untertriage als akzeptabler angesehen“ werde. Dies erscheint plausibel, wenn man das Outcome untertriagierter Traumapatient:innen betrachtet. Studien zeigen, dass eine Untertriage mit einem erhöhten Risiko für Morbidität und Mortalität einhergeht. Tignanelli et al. [12] berichten beispielsweise von einer Mortalität von 30 % bei Patient:innen, die zwar ein American College of Surgeons (ACS)-Aktivierungskriterium erfüllen, aber keiner vollständigen Traumateambehandlung zugeführt wurden, lag die Mortalität bei korrekt triagierten Patient:innen bei 21 %, bei Patient:innen ohne TTA-Kriterien nur bei 4 % [12]. In der eigenen Arbeit zeigt sich eine deutlich geringere Mortalität der untertriagierten Patient:innen. Es gab eine geringe Anzahl an untertragierten Patient:innen, jeweils ohne letalen Verlauf. Eine Untertriage hatte in der aktuellen Studie bei niedrigen Fallzahlen also keinen negativen Einfluss auf das Outcome der Patient:innen. Der Vergleich der beiden Analysen ist jedoch aufgrund der unterschiedlichen TTA-Indikationsstellungen sowie der international gängigeren Abstufung zwischen kompletten oder reduzierten Aktivierungen von Traumateams, nur eingeschränkt möglich.

Im internationalen Konsens gilt eine Übertriage von bis zu 50 % als akzeptabel, um eine Untertriage von < 5 % sicherzustellen. Als Zielkorridor wird jedoch eine Übertriage von lediglich 25–35 % empfohlen [11]. Ein Wert, der in der vorliegenden Studie mit 70,8 % deutlich verfehlt wurde. Obwohl das Übertriageziel somit nicht erreicht wurde, lag die potenziell gefährdende Untertriage klar unter dem geforderten Grenzwert.

Ob sich in einzelnen Studien erstmalig dokumentierte Verknüpfungen von objektiven, automatisiert übermittelten Unfalldaten mit Behandlungsdaten sowie dem TR-DGU® auf die Triagierung auswirken bleibt abzuwarten. Möglicherweise können sich unfallmechanismusspezifische Erkenntnisse, z. B. durch die unterschiedliche Bewertung von frontalen zu seitlichen Krafteinwirkungen bei Unfallereignissen, auf die Triage und ggf. auch die TTA auswirken [14].

### Verletzungsschwere, Verweildauer und Mortalität

Unter dem Aspekt, dass die HRSI- und MRSI-Kriterien in der aktualisierten S3-Leitlinie Polytrauma/Schwerverletzten-Behandlung die Treffsicherheit der TTA verbessern sollen, erscheint es wichtig auszuschließen, dass sich die Schwere der Verletzung oder die Mortalität der Patient:innen verändert haben. In Bezug auf die Verletzungsschwere und die Gesamtverweildauer der schwerverletzten Patient:innen ließen sich trotz kürzer erscheinender Aufenthaltsdauer der Post-Leitlinienkohorte keine signifikanten Unterschiede feststellen. Ein multizentrischer Vergleich, z. B. über das TR-DGU® bleibt diesbezüglich abzuwarten, kann jedoch erst im langfristigen Verlauf erfolgen.

Ein ähnliches Bild ergibt sich bei Betrachtung der Mortalität. In beiden Kohorten verstarben je etwa 1 % der Patient:innen. Diese wurden stets primär rot triagiert und folgerichtig über den Schockraum vorgestellt. Auch die mittlere intensivmedizinische Behandlungsdauer vor dem Versterben und die ISS-Werte der verstorbenen Patient:innen unterscheiden sich zwischen den Kohorten nicht signifikant. Die Tatsache, dass keine Todesfälle außerhalb der Schockraumgruppe auftraten, könnte als Hinweis gewertet werden, dass es zu keiner wesentlichen Untertriage kam.

### Limitationen

Trotz prospektiver Datenerhebung und der klinischen Relevanz unter Alltagsbedingungen weist die vorliegende Studie mehrere Limitationen auf.

Es handelt sich um eine monozentrische Untersuchung an einem einzigen ÜTZ. Die Auswertung schließt nicht alle Schockraumindikatoren mit ein. Sie bezieht sich nur auf Indikationen, die nach Unfallereignissen mit Kraftfahrzeugen getroffen wurden.

Daraus ergibt sich eine eingeschränkte Übertragbarkeit der Ergebnisse auf andere Versorgungsregionen und Klinikstrukturen. Unterschiede in der regionalen Verteilung von Unfallarten, präklinischen Abläufen, Klinikgröße, personellen Ressourcen und Implementierung der Leitlinie können die TTA und deren Anzahl maßgeblich beeinflussen.

Die Veröffentlichung der aktuellen S3 Leitlinie Polytrauma‑/Schwerverletzten-Behandlung im Februar 2023 erfolgte kurz nach dem Jahreswechsel. Da der Termin der Veröffentlichung unbekannt war, könnten die Jahresplanungen für Fortbildungen und Prozessanpassung im klinischen und präklinischen Bereich ggf. bereits abgeschlossen gewesen sein. Es ist davon auszugehen, dass die Novellierung der Kriterien insbesondere in Krankenhäusern mit Zuweisungen mehrerer Rettungsdienste in den ersten Monaten nach Inkrafttreten noch nicht flächendeckend zur Anwendung kam. Die Auswirkung der LA kann somit aufgrund fehlender Schulungen geringer ausgeprägt sein als im langfristigen Verlauf.

Die Studie konzentrierte sich primär auf Struktur- und Prozessparameter wie Triagekategorie, Schockraumaufnahme und Verweildauer. Eine detaillierte Analyse der Positionen der Personen während des Unfallereignisses (z. B. inner- oder außerhalb eines PKW) und auch klinischer Parameter wurden nicht durchgeführt. Auch wurde nicht nachvollzogen, ob der ambulanten Behandlung eine Entlassung gegen ärztlichen Rat vorausging, oder Untersuchungen verweigert wurden. Dies limitiert die Aussagekraft hinsichtlich der tatsächlichen Unfallmechanismen und Behandlungsverläufe.

Eine Erhebung der ISS erfolgte ausschließlich bei Patient:innen, die im TR-DGU® dokumentiert wurden. Für alle anderen Fälle erfolgte keine standardisierte Einschätzung der Verletzungsschwere. Hierdurch ist die Vergleichbarkeit der Gruppen eingeschränkt.

## Fazit für die Praxis

- Die vorliegende Studie konnte nachweisen, dass durch die Novellierung der S3-Leitlinie „Polytrauma/Schwerverletzten-Behandlung“ die Übertriage nach Verkehrsunfällen bereits kurzfristig signifikant reduziert wurde, ohne dass es zu einer Zunahme an untertriagierten Patient:innen kam.
- Diese Ergebnisse untermauern, dass die Entfernung der MRSI-Kriterien („moderate risk for severe injury“) eine sichere und zielgerichtete Traumateamaktivierung (TTA) ermöglichen, ohne das Risiko für schwerverletzte Patient:innen zu erhöhen.
- Zur Bestätigung der Ergebnisse und zum Ausschluss lokaler Besonderheiten sind multizentrische Studien incl. einer strukturierten Implementierung der Leitlinienkriterien in Klinik und Präklinik anzustreben. Künftige Studien sollten zudem den Unfallmechanismus einbeziehen und dabei auch die Position inner- oder außerhalb des Fahrzeugs als Fußgänger:in oder Radfahrer:in berücksichtigen.

## Data Availability

Die Datensätze, die dieser Studie zugrunde liegen, sind auf begründete Anfrage beim korrespondierenden Autor erhältlich.

## Abkürzungen

ACS: American College of Surgeons
ACS-COT: American College of Surgeons Committee on Trauma
Delta‑V: Geschwindigkeitsänderung im Rahmen eines Unfallereignisses
DGU®: Deutsche Gesellschaft für Unfallchirurgie e. V.
DGUV: Deutsche Gesetzliche Unfallversicherung
GCS: Glasgow Coma Scale
HRSI: „High risk for severe injury“
ISS: Injury Severity Score
KISS: Krankenhausinformationssystem
LA: Leitlinienaktualisierung
mm Hg: Millimeter Quecksilbersäule
MRSI: „Moderate risk for severe injury“
MTS: Manchester Triage System
MW: Mittelwert
MWU: Mann-Whitney-U-Test
Post-LA: Post-Leitlinienaktualisierung
Prä-LA: Prä-Leitlinienaktualisierung
S3: Systematische Leitlinie Stufe 3
SAV: Schwerverletztenverfahren
SD: Standardabweichung
SpO_2_: Sauerstoffsättigung
TR-DGU®: TraumaRegister der Deutschen Gesellschaft für Unfallchirurgie
TTA: Traumateamaktivierung
ÜTZ: Überregionales Traumazentrum
ZNA: Zentrale Notaufnahme
